# Parameter estimation in a Holzapfel–Ogden law for healthy myocardium

**DOI:** 10.1007/s10665-014-9740-3

**Published:** 2015-01-30

**Authors:** H. Gao, W. G. Li, L. Cai, C. Berry, X. Y. Luo

**Affiliations:** 1 School of Mathematics and Statistics, University of Glasgow, Glasgow, UK; 2 School of Science, Northwestern Polytechnical University Xi’an, Xi’an, 710072 Shaanxi People’s Republic of China; 3 Institute of Cardiovascular and Medical Sciences, University of Glasgow, Glasgow, UK

**Keywords:** Holzapfel–Ogden law, Inverse problem, Left ventricular, Parameter estimation, Passive myocardial properties

## Abstract

A central problem in biomechanical studies of personalized human left ventricular (LV) modelling is to estimate material properties from in vivo clinical measurements. In this work we evaluate the passive myocardial mechanical properties inversely from the in vivo LV chamber pressure–volume and strain data. The LV myocardium is described using a structure-based orthotropic Holzapfel–Ogden constitutive law with eight parameters. In the first part of the paper we demonstrate how to use a multi-step non-linear least-squares optimization procedure to inversely estimate the parameters from the pressure–volume and strain data obtained from a synthetic LV model in diastole. In the second part, we show that to apply this procedure to clinical situations with limited in vivo data, additional constraints are required in the optimization procedure. Our study, based on three different healthy volunteers, demonstrates that the parameters of the Holzapfel–Ogden law could be extracted from pressure–volume and strain data with a suitable multi-step optimization procedure. Although the uniqueness of the solution cannot be addressed using our approaches, the material response is shown to be robustly determined.

## Introduction

Computational modelling of left ventricular (LV) mechanics provides unique insights into LV functions in both diseased and healthy states. Such modelling relies essentially on the knowledge of constitutive laws and the corresponding myocardial material properties [1]. These material properties also provide invaluable diagnostic information for patient risk stratification [2]. For example, the passive mechanical stiffness of the myocardium can affect the diastolic filling of LV, which further affects the systolic pump function. Estimating the constitutive parameters non-invasively, however, remains a great challenge for the LV modelling community.

Applications of the finite-element method (FEM) in biomechanical LV modelling date back to the 1970s with homogeneous isotropic linear material [3–5] in diastole, then followed by three-dimensional stress analysis in the myocardial wall of human LV reconstructed from clinical data [6–8]. By considering the left ventricle of a beating dog heart as a thick-walled, fibre-reinforced structure and using non-linear finite-element analysis, Guccione et al. [9] simulated LV dynamics at end of diastole (ED) and end of systole (ES) with a transversely isotropic constitutive law [10]. Recently, the FEM has been applied to patient-specific modelling of heart diseases, and the models may consist of anatomic reconstruction, electrical activity, biomechanics and haemodynamics with non-linear hyper-elastic constitutive laws [11, 12]. Note that in those investigations, one solves either the static or the dynamic problems with the given material properties.

Traditionally, the mechanical properties of the LV myocardium are determined by a series of uni-axial [13] or biaxial sample tests [14] or simple shear deformations [15] on specimens harvested from a specific heart. These experiments provide insights into not only formulating a constitutive law of the myocardium as well as determining the parameters [16]. However, these methods involve invasive operations and the destruction of tissues and are not suitable for in vivo or clinical applications.

An alternative method of determining passive LV mechanical behaviour is the inverse estimation. For example, parameters of a forward problem can be tuned to match the pressure–volume and motion fields provided by clinical imaging. This approach was first used in [17, 18]. The inverse estimation is typically formulated as a non-linear optimization problem to minimize the difference in the measurements with respect to the unknown parameters. Because of the highly non-linear nature of the optimization problem, and because that the constitutive parameters are often correlated, it is non-trivial to inversely estimate those parameters accurately and uniquely from the noised measurements [19], especially when complicated constitutive laws are used [20].

Earlier studies mainly used homogeneous isotropic linear elastic material models for the myocardium, which are inappropriate in modelling myocardial mechanical behaviour since it is a hyper-elastic, fibre-reinforced material; for summary on the myocardial material models, the interested reader is referred to [1]. Guccione et al. [10] inversely estimated the parameters of a non-linear, transversely isotropic, four-parameter strain energy function (known as Guccione’s law) using a three-dimensional (3D) model reconstructed at the beginning of the diastole. The inverse method was later extended to include passive 3D LV aneurysm properties [21–24]. Augenstein et al. [25, 26] described an ex vivo experimental method and apparatus for myocardial parameter estimation using cardiac magnetic resonance (CMR) imaging and Guccione’s law. The sequential quadratic programming (SQP) method was used to optimize parameters by matching the experimentally measured geometry, deformation fields and applied boundary conditions. The method was also validated against a deformable silicon gel phantom with known material properties. Their results suggested that it is feasible to extract meaningful biophysical parameters from CMR data. Nair et al. [27] used a genetic algorithm to estimate the four parameters of Guccione’s law with a rabbit LV model by matching the strains.

Using in vivo CMR images combined with ex vivo diffusion tensor CMR, Wang et al. [28] sequentially estimated the four parameters of Guccione’s law on a canine model using motion data from two states of the left ventricle: unloaded reference state and ED. SQP was used for the optimization procedure, too. Later the researchers applied the method to estimate the in vivo myocardial tissue properties on heart failure patients [29]. Because of a lack of motion data, only one parameter from Guccione’s law was optimized by matching the LV dynamics; the remaining three parameters were taken from canine studies. Xi et al. [30] used a reduced-order unscented Kalman filter to optimize Guccione’s law from an in vivo CMR study for a human heart. They also estimated relaxation parameters in diseased LV models [2] by combining cine and tagged CMRs along with invasively measured ventricular pressure.

The aforementioned studies all used Guccione’s law, which assumes that the myocardium is transversely isotropic. Recent studies have demonstrated that myofibres have a highly laminar structure forming local orthotropic material axes inside the myocardium [31]. It has been pointed out by Schmid et al. [32] that a transversely isotropic law is not suitable for modelling the passive myocardial response in simple shear tests. Therefore, to account for the layered micro-structure, orthotropic constitutive laws have been proposed, such as the Fung-type law [33], pole-zero law [34] and strain-invariant-based law [1]. Generally, there are more parameters in orthotropic constitutive laws compared to transversely isotropic laws, which makes the inverse estimation harder to do. Using a synthetic LV model, Remme et al. [20] inversely estimated parameters for the pole-zero law. However, because of the high intercorrelations among the parameters, only 3 out of 18 parameters were estimated.

Although a number of studies have demonstrated the feasibility of inversely estimating constitutive parameters from in vivo clinical measurements using simpler constitutive relations [10], fewer attempts have been made to estimate parameters from in vivo data using orthotropic laws [20]. The orthotropic constitutive law proposed in [1] (known as the Holzapfel–Ogden law) has strong ellipticity with respect to material stability and fewer parameters than the pole-zero law. Most importantly, the advantage of the Holzapfel–Ogden law is that it can account for a layered myofibre architecture. The Holzapfel–Ogden law is also relatively easy to implement using the FEM. However, to the authors’ best knowledge, the feasibility of identifying the parameters of the Holzapfel–Ogden law from non-invasive clinical measurements has not yet been investigated.

In this paper, we carry out such a study for the first time using a previously published LV model with known parameters to provide a set of synthetic strain data and pressure–volume relationships [35]. Once verified, the optimization method is then extended and applied to in vivo models with limited clinical measurements.

## Methodology

### Constitutive law for passive myocardium

The Holzapfel–Ogden constitutive law [1] assumes that the strain energy function $\psi$ for the myocardium is

1 $$\begin{aligned} \psi = \frac{a}{2b}\mathrm{e}^{b(I_1-3)}+ \sum _{i=\text {f},\text {s}} \frac{a_i}{2b_i}[\mathrm{e}^{b_i(I_{\text {4i}}-1)^2}-1] + \frac{a_\text {fs}}{2b_\text {fs}}[\mathrm{e}^{b_\text {fs}I_\text {8fs}}-1], \end{aligned}$$

where $a,\,b$ are the parameters for the matrix response; $a_\text {f},\,b_\text {f}$ are the parameters for the myocardial fibres; $a_\text {s}$ and $b_\text {s}$ account for the fibre sheet contribution; and $a_\text {fs},\,b_\text {fs}$ represent the shear effects in the sheet-plane. $I_1=\text{ tr }(\mathbf{C}),\,\mathbf{C}=\mathbf{F}^\mathrm{T}\mathbf{F}$ is the right Cauchy–Green tensor, and $\mathbf{F}$ is the deformation gradient. $\hbox {I}_\mathrm{4f} = \mathbf{f_{0}} \cdot (\mathbf{C}\mathbf{f_{0}})$ and $I_\mathrm{4s} = \mathbf{s_{0}} \cdot (\mathbf{C}\mathbf{s_{0}})$ are the stretch-related invariants along the myocyte and sheet directions, $\mathbf{f}_0$ and $\mathbf{s}_{0}$ respectively, in the reference configuration. $I_\mathrm{8fs} = \mathbf{f_0} \cdot (\mathbf{C}\mathbf{s_0})$ reflects the coupling between the fibre and sheet stretches. For a more detailed description of the Holzapfel–Ogden law, please refer to [1] and, for its applications in LV modelling, to [35–39].

### Study 1: Feasibility study

#### Synthetic model

To check the feasibility of the inverse estimation of parameters in the Holzapfel–Ogden law from strain and pressure–volume data, a previously published passive LV model [35] with known material parameters (i.e. the so-called true parameters) is simulated using ABAQUS FEA. The myofibre structure and boundary conditions of the LV model are kept the same as in [35]. This produces a set of synthetic data (i.e. the so-called experimental data) when the endocardial pressure is increased from 0 to 8 mmHg. From these data we extract the LV cavity volume, as well as the first, second and third principal strains from randomly distributed observation points inside the LV wall (excluding the basal plane where the boundary conditions are applied) throughout the LV diastolic loading phase.

#### Optimization procedure

We define three objective functions for matching the experimental data:

2 $$\begin{aligned} \begin{array}{ll} &{} f_\mathrm{obj} = f_\mathrm{vol} + f_{\varepsilon }, \\ &{} f_\mathrm{vol} = \sum \limits _{i=1}^{T^N_\text {exp}}\Big (1-\dfrac{V_{i}}{V_{i}^{*}}\Big )^2, \\ &{} f_{\varepsilon } = \sum \limits _{i=1}^{T^N_\text {exp}} \sum \limits _{j=1}^{N_\text {point}} \sum \limits _{k=\text {1st, 2nd, 3rd}}(\varepsilon ^k_{j,i}-\varepsilon _{j,i}^{k\text {*}})^2, \\ \end{array} \end{aligned}$$

where $T^N_\text {exp}=25$ is the number of timesteps from the beginning to the end of pressure loading; $N_\text {point}=20$ is the number of observation points randomly distributed within the LV wall; $V_{i}^\text {*}$ and $\varepsilon _{j,i}^{k\text {*}}$ are the LV chamber volume and principal strains at timestep $i$ from the forward modelling; $V_{i}$ and $\varepsilon ^k_{j,i}$ are the corresponding data from the inverse procedure; and $k$ indicates the first, second and third principal strains.

A sensitivity analysis of parameters is performed by varying each of the eight parameters in a range of $\pm$10% around their so-called true values, while the seven remaining parameters are chosen to be the true values. A study of the sensitivity of the objective functions to the variation of a parameter is then performed and summarized in Table 1. Notice that because the total number of data points for the volume (25) is much less than the total number of the strains (1,500), the change ${\varDelta } f_{\varepsilon }$ and, hence, ${\varDelta } f_\mathrm{obj}$ is much greater than ${\varDelta } f_\mathrm{vol}$. In addition, $f_\mathrm{obj},\,f_\mathrm{vol}$ and $f_\varepsilon$ are most sensitive to changes in $a_\text {f}$, followed by $a,\,b,\,a_\text {fs}$, but are less sensitive to changes in $a_\text {s}$ and $b_\text {s}$. This suggests that $a_\text {s}$ and $b_\text {s}$ may not be accurately estimated. However, a greater change in $f_\mathrm{vol}$ due to the variation in $b_\text {f}$ is observed; thus, using $f_\mathrm{vol}$ in addition to $f_{\varepsilon }$ allows one to extract extra information.

**Table 1:** Changes in objective function for each parameter varied by $\pm$10% from original values, defined as ${\varDelta } O = (O^{+} +O^{-})/2$; $O^+$ is the change from a $+$10% increase in one parameter, and $O^-$ is the change from a $-$10% decrease in one parameter

	$a$ (kPa)	$b$	$a_\text {f}$ (kPa)	$b_\text {f}$	$a_\text {s}$ (kPa)	$b_\text {s}$	$a_\text {fs}$ (kPa)	$b_\text {fs}$
${\varDelta } f_\mathrm{obj}$	0.006	0.004	0.014	0.0008	0.0004	0.0004	0.003	0.0008
${\varDelta } f_\mathrm{vol}$	$8.7 \times 10^{-6}$	$6.9 \times 10^{-6}$	0.0009	$2.1 \times 10^{-5}$	$3.6 \times 10^{-7}$	$3.6 \times 10^{-7}$	$2.2 \times 10^{-6}$	$7.1 \times 10^{-7}$
${\varDelta } f_{\varepsilon }$	0.006	0.004	0.013	0.0007	0.0004	0.0004	0.003	0.0008

**Fig. 1:**
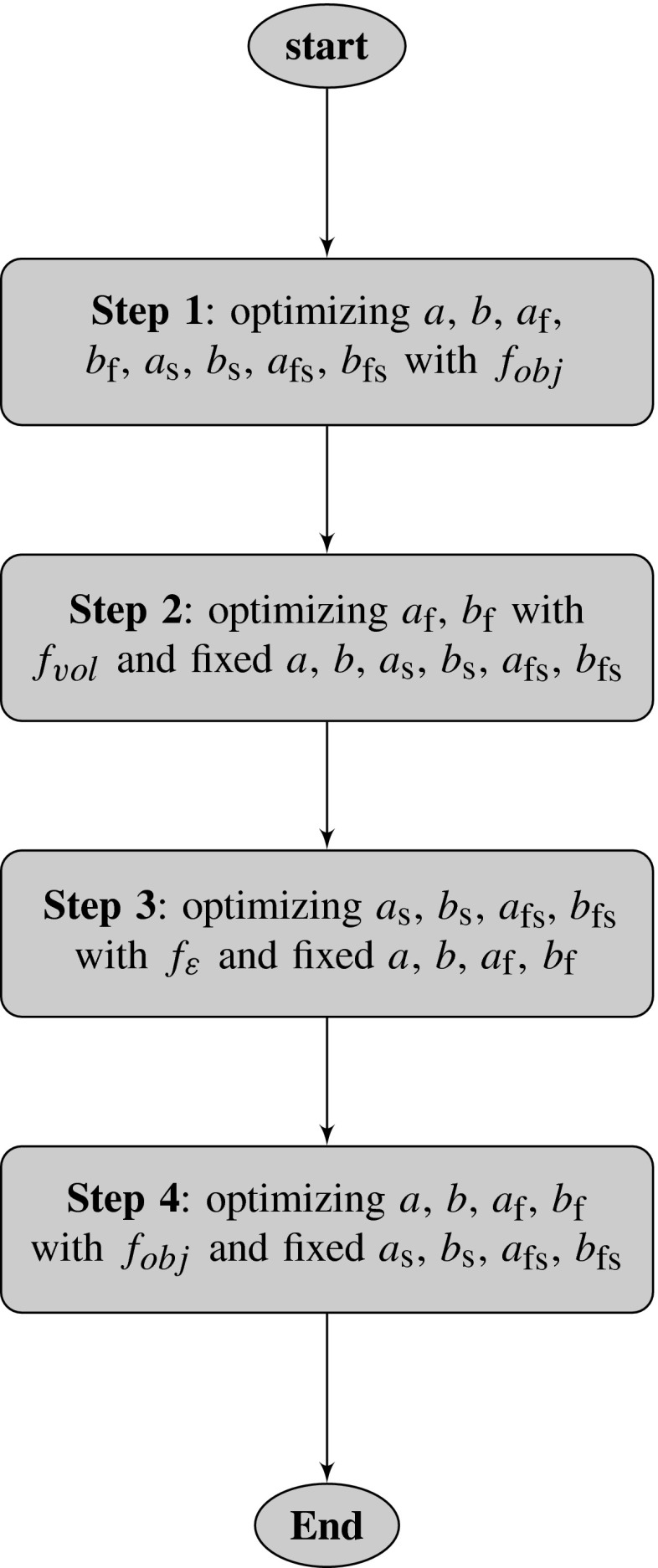
Flowchart of proposed multi-step optimization procedure for the synthetic model. In Step 1, all the parameters are estimated using both the volume and strain data, followed by (Step 2) an estimation of $a_\text {f}$ and $b_\text {f}$ using the volume data only. Similarly in Step 3, $a_\text {s},\,b_\text {s},\,a_\text {fs},\,b_\text {fs}$ are estimated using the strain data only. In Step 4, the remaining parameters $a,\,b,\,a_\text {f},\,b_\text {f}$ from Step 3 are optimized using both the volume and strain data

Based on the sensitivity analysis, we propose a multi-step optimization procedure, as shown in Fig. 1. In each step, a forward problem is solved to yield the updated data set $V_i$ and $\varepsilon ^{k}_{j,i}$. The MATLAB function lsqnonlin, together with a trust-region-reflective algorithm, is used to update the material parameters by minimizing the least-squares difference between the experimental data and the model responses from the forward simulations.

### Study 2: Application to in vivo modelling

**Fig. 2:**
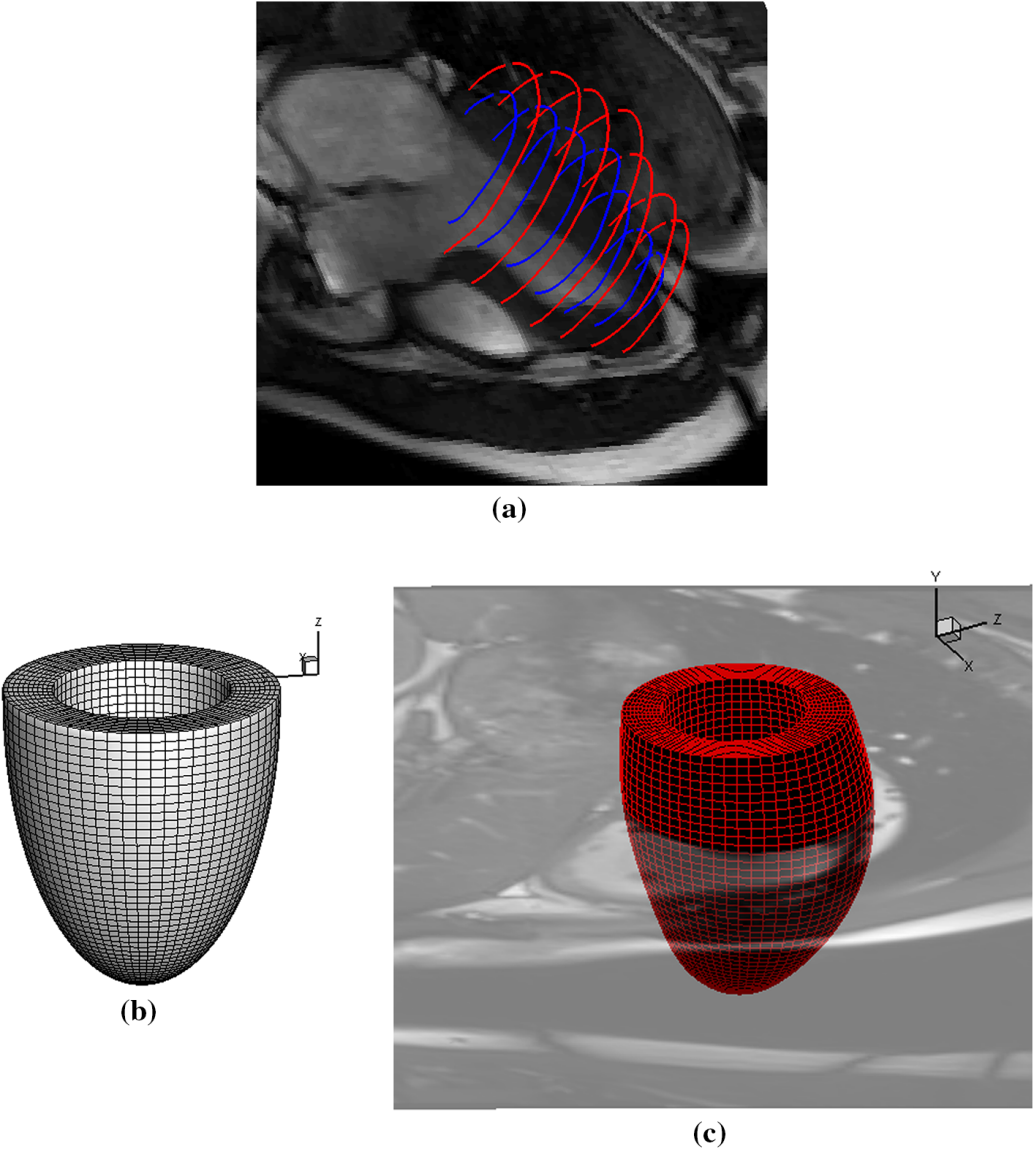
Computational in vivo LV model. **a** Endocardial and epicardial boundaries segmentation. **b** Reference LV mesh described in prolate spheroidal coordinates. **c** Fitted LV mesh

Having developed the multi-step optimization procedure, we proceed to estimate the parameters of the Holzapfel–Ogden law using in vivo data. An in vivo LV geometry is first reconstructed from a CMR study of a healthy male volunteer, age 28. The CMR study was performed on a Siemens MAGNETOM Avanto (Erlangen, Germany) 1.5 Tesla scanner with a 12-element phased array cardiac surface coil. The imaging protocol included cine sequence with steady-state free precession. Custom MATLAB software was written to segment the endocardial and epicardial boundaries at early diastole when the LV pressure is lowest (Fig. 2a). Following the manual segmentation, prolate spheroidal coordinates and cubic B-splines are used to fit endocardial and epicardial surfaces, smoothness regularization is imposed to minimize the geometric distortion, and constraints are imposed on the fitting parameters to maintain $C^2$ continuity at the apex. Figure 2b shows the unfitted reference LV geometry. Hexahedral elements are generated using a linear interpolation from endocardial to epicardial surfaces, as shown in Fig. 2c. The interested reader is referred to [40] for more details on the LV geometry reconstruction.

An in-house B-spline deformable registration method [41] is used to estimate the regional circumferential strain from early to ED from four positions of short-axis cine images from basal to middle ventricles and six regions for each short-axis position. The LV cavity volumes at ED are also calculated from the cine images. In summary, the data from the in vivo measurements consist of 24 regional circumferential strains and LV cavity volume at ED. Because the ventricular pressure recording is not available, a population-based ED pressure (8 mmHg) is assumed.

#### Sensitivity analysis

Generally, the in vivo measurements are more noisy and contain significantly fewer data compared to what can be produced by the synthetic model (25 versus 1,525), so a more comprehensive sensitivity analysis is performed.

Consider a parameter set $\{\kappa _i, i=1,\ldots 8\}:= [a, b, a_\text {f}, b_\text {f}, a_\text {s}, b_\text {s}, a_\text {fs}, b_\text {fs}]$, and define

3 $$\begin{aligned} \{d_i, i=1, \ldots , N \} = \{ \varepsilon _1,\ldots , \varepsilon _n, V \}, \end{aligned}$$

where $N = n+1,\,\varepsilon _i$ is the strain data from the $i$th position ($i=1,\ldots ,n$), and $V$ is the LV volume. To measure the model response sensitivity to parameter variations ($10\%$ as in Sect. 2.2.2), we introduce the matrix

$$\begin{aligned} S = \left( \begin{array}{cccc} \displaystyle \frac{{\varDelta } d_1}{{\varDelta } \kappa _1} &{} \quad \displaystyle \frac{{\varDelta } d_1}{{\varDelta } \kappa _2} &{} \quad \displaystyle \ldots &{} \quad \displaystyle \frac{{\varDelta } d_1}{{\varDelta } \kappa _8} \\ \displaystyle \frac{{\varDelta } d_2}{{\varDelta } \kappa _1} &{} \quad \displaystyle \frac{{\varDelta } d_2}{{\varDelta } \kappa _2} &{} \quad \displaystyle \ldots &{} \quad \displaystyle \frac{{\varDelta } d_2}{{\varDelta } \kappa _8} \\ \displaystyle \vdots &{} \quad \displaystyle \vdots &{} \quad \displaystyle \ddots &{} \quad \displaystyle \vdots \\ \displaystyle \frac{{\varDelta } d_N}{{\varDelta } \kappa _1} &{} \quad \displaystyle \frac{{\varDelta } d_N}{{\varDelta } \kappa _2} &{} \quad \displaystyle \ldots &{} \displaystyle \frac{{\varDelta } d_N}{{\varDelta } \kappa _8} \end{array} \right) , \end{aligned}$$

where the relative changes in the strains and volume due to the parameter variations ${{\varDelta } \kappa _m}$ are denoted by ${\varDelta } d_{i}={\varepsilon _{i} - \varepsilon _{i0}}, i \in N, 0$ indicates the data from the baseline model. If the variations in any of the parameters produce similar responses, then these parameters may be correlated. To quantify the correlations, we further calculate the sensitivity coefficient matrix SCM from the normalized columns of $S$:

4 $$\begin{aligned} \mathrm{SCM} =[\bar{S}_m]^T [\bar{S}_m] \,\hbox { and }\, m=1,\ldots ,8, \;\;{\bar{S}}_m=\frac{S_m}{|S_m|}, \;\; S_m=\left\{ \frac{{\varDelta } d_{i}}{{\varDelta } \kappa _m}, i=1,\ldots N\right\} . \end{aligned}$$

If $\mathrm{SCM}_{i,j}$ is close to $\pm 1$, then $\kappa _i$ and $\kappa _j$ are closely correlated. The existence of correlated parameters poses non-uniqueness in the inverse problem. A possible remedy is to estimate one parameter only while leaving the correlated ones constant. In addition, the norm

$$\begin{aligned} |S_m|=\sqrt{\sum ^N_i\left( \frac{{\varDelta } d_{i}}{{\varDelta } \kappa _m}\right) ^2} \end{aligned}$$

reflects the sensitivity of the objective function to the variation of each parameter. Low sensitivity to a parameter makes it difficult to estimate.

Table 2 lists the average results of two sensitivity analyses: one is from a $10\%$ increase in each parameter, the other is from a $10\%$ decrease in each parameter; the baseline parameters are from [36]. These results show that $a$ has the highest sensitivity, followed by $a_\text {f},\,a_\text {fs},\,b_\text {f},\,b,\,b_\text {fs}$. $a_\text {s}$ has a much lower sensitivity, and $b_\text {s}$ has virtually zero sensitivity. High correlations can also be found among $a,\,b,\,a_\text {f},\,b_\text {f},\,a_\text {s},\,a_\text {fs},\,b_\text {fs}$. These observations suggest that it is unlikely that the eight parameters can be estimated unambiguously.

**Table 2:** Correlation coefficient SCM with LV volume

	$a$	$b$	$a_\text {f}$	$b_\text {f}$	$a_\text {s}$	$b_\text {s}$	$a_\text {fs}$	$b_\text {fs}$
$a$	1.0000	1.0000	0.9999	0.9998	0.9570	$\approx 0.0$	0.9999	1.0000
$b$		1.0000	0.9999	0.9998	0.9571	$\approx 0.0$	0.9999	1.0000
$a_\text {f}$			1.0000	1.0000	0.9572	$\approx 0.0$	0.9997	0.9999
$b_\text {f}$				1.0000	0.9572	$\approx 0.0$	0.9997	0.9999
$a_\text {s}$					1.0000	0.1	0.9568	0.9570
$b_\text {s}$						1.0000	$\approx 0.0$	$\approx 0.0$
$a_\text {fs}$							1.0000	0.9999
$b_\text {fs}$								1.0000
Sensitivity values								
	50.6839	2.9955	8.7953	3.3863	0.0001	$\approx 0.0$	6.9506	0.2714

Since the absolute LV volume is much greater than the strains, it can overdominate the optimization procedure. Hence, we also perform the same sensitivity study using the normalized LV volume, $({V-V_0})/{V_0}$, and the results are shown in Table 3. Now the correlation matrix SCM is different from that in Table 2. The four parameters $a,\,b,\,a_\text {fs},\,b_\text {fs}$ are highly correlated, while $a_\text {f}$ and $b_\text {f}$ are only correlated to each other, not to $a,\,b,\,a_\text {fs},\,b_\text {fs}$. Again, $a_\text {s}$ and $b_\text {s}$ have very low sensitivity to parameter variations.

**Table 3:** Correlation coefficient SCM with normalized LV volume

	$a$	$b$	$a_\text {f}$	$b_\text {f}$	$a_\text {s}$	$b_\text {s}$	$a_\text {fs}$	$b_\text {fs}$
$a$	1.0000	0.9918	$-0.0505$	$-0.1202$	$-0.01$	0.02	0.9155	0.8590
$b$		1.0000	$-0.0940$	$-0.1600$	$-0.002$	0.02	0.8865	0.7985
$a_\text {f}$			1.0000	0.9952	0.06	$-0.006$	$-0.2722$	0.2759
$b_\text {f}$				1.0000	0.04	$\approx 0.0$	$-0.3442$	0.2026
$a_\text {s}$					1.0000	0.004	$-0.02$	$-0.004$
$b_\text {s}$						1.0000	0.004	$\approx 0.0$
$a_\text {fs}$							1.0000	0.8296
$b_\text {fs}$								1.0000
Sensitivity values								
	0.5953	0.0367	0.0960	0.0389	$\approx 0.0$	$\approx 0.0$	0.1373	0.0028

To deal with the difficulties caused by the correlation of the parameters, we follow the approach by [2] and divide the parameters into two groups, $a^\text {group} = \{a, a_\text {f}, a_\text {s}, a_\text {fs}\},\,b^\text {group} = \{b, b_\text {f}, b_\text {s}, b_\text {fs}\}$. We set

5 $$\begin{aligned} \begin{array}{l} a^\text {group} = C_a a^\text {group}_0,\quad b^\text {group} = C_b b^\text {group}_0, \end{array} \end{aligned}$$

where $a^\text {group}_0$ and $b^\text {group}_0$ are the experimentally estimated parameters from the previous study [35] (referred to as the original parameters), and $C_a,\,C_b$ are the scaling factors.

In the first optimization step, the optimal $C_a$ and $C_b$ are found by minimizing

6 $$\begin{aligned} f_{O1} = \sum _{i=1,\ldots ,n}{(\varepsilon _i -\varepsilon _i^*)^2} + (V-V_0)^2, \end{aligned}$$

in which, $\varepsilon _i^*$ is the measured regional circumferential strain and $V_0$ is the measured LV cavity volume from cine images. The minimization problem is solved by sweeping the parameter space ($C_a$ and $C_b$) as in [2]. The final parameter set which minimizes $f_{O1}$ is chosen. If multiple sets of $C_a$ and $C_b$ exist, the set with the minimized $|C_a -C_b|$ is selected.

Because $f_{O1}$ is volume dominated, further steps are needed to match the strains. According to Table 3, $a_\text {f}$ is only highly related to $b_\text {f}$, but not to $a,\,b,\,a_\text {fs}$ and $b_\text {fs}$; therefore, we could set $a,\,b,\,a_\text {fs}$ and $b_\text {fs}$ at the same values from the first step but optimize $a_\text {f},\,b_\text {f}$ with the following objective function:

7 $$\begin{aligned} f_{O2} = \sum _{i=1,\ldots ,n}{(\varepsilon _i -\varepsilon _i^\text {*})^2} + \Big (\frac{V-V_0}{V_0}\Big ) ^2. \end{aligned}$$

However, since the sensitivities of $a_\text {s}$ and $b_\text {s}$ are very low, the optimization procedure is still unable to update these without further information.

In a healthy myocardium, we know that the stiffness in the sheet direction should be much smaller than that in the myofibre direction; hence, we introduce the following constraints:

8 $$\begin{aligned} a_\text {f}\ge 2a_\text {s}\quad \text{ and } \quad b_\text {f}\ge 2b_\text {s}, \end{aligned}$$

in the optimization procedure to allow $a_\text {s}$ and $b_\text {s}$ to be updated. The SQP method in MATLAB is then employed to minimize $f_{O2}$ with the constraints (Eq. 8).

A further step is used to ensure that the volume matching is still satisfied by applying the objective function $f_{O1}$ once more. Since Table 2 indicates that $f_{O1}$ is highly sensitive to variations in $a$ and $a_\text {fs}$, which are also correlated, we may reduce one of these two parameters by introducing $C_3$, so that

9 $$\begin{aligned} a = C_3 a \quad \text{ and } \quad a_\text {fs} = C_3 a_\text {fs}. \end{aligned}$$

Now we only need to estimate $C_3$ in the third step. The remaining parameters are kept the same from Step 2.

**Fig. 3:**
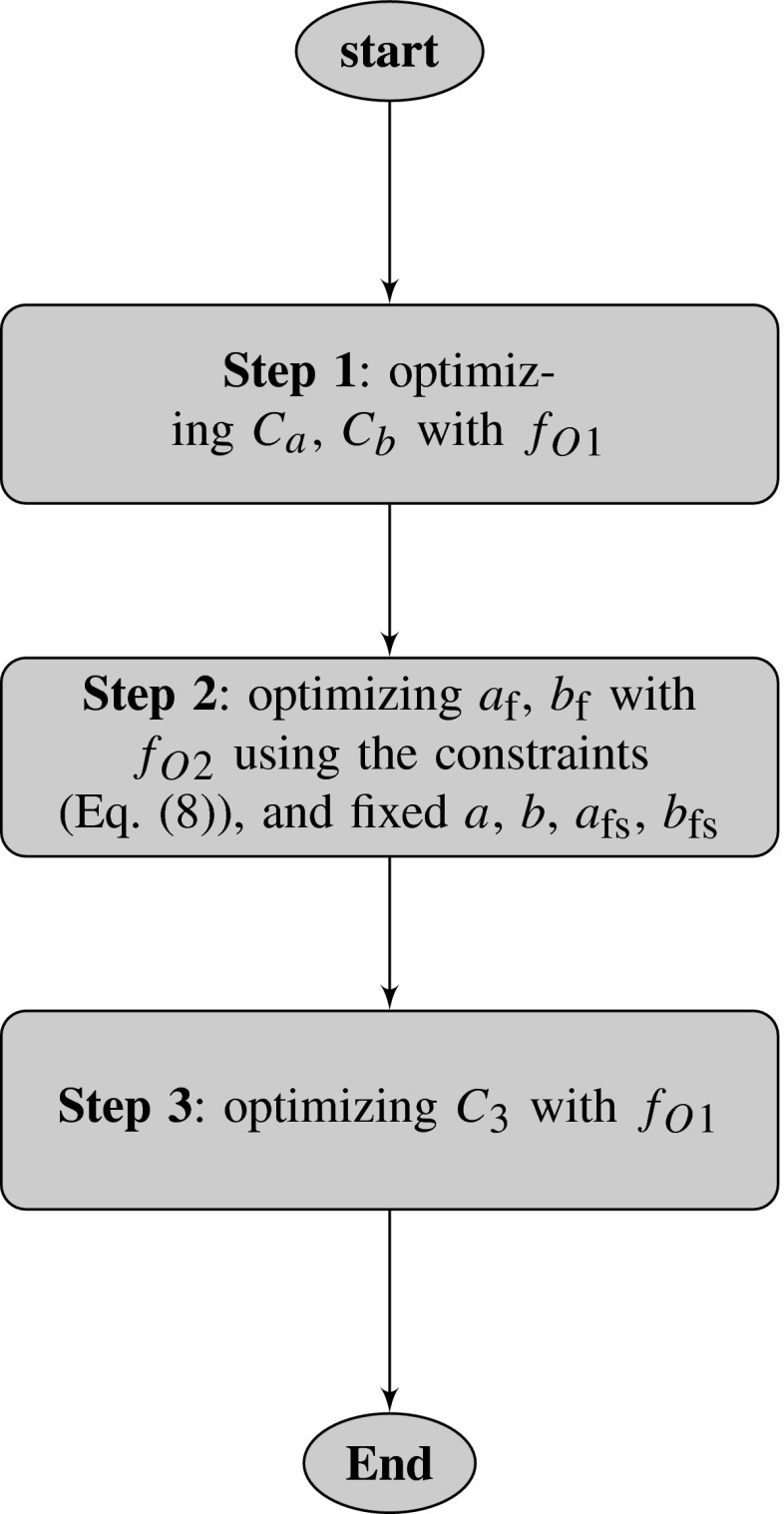
Flowchart of proposed multi-step optimization procedure for in vivo LV model. In Step 1, all parameters from the Holzapfel–Ogden law are updated through the two scaling factors $C_a$ and $C_b$ (Eq. 5) by minimizing $f_{O1}$ (Eq. 6), followed by (Step 2) the optimization of $a_\text {f}$ and $b_\text {f}$ by minimizing $f_{O2}$ (Eq. 7). Finally, in Step 3, $a$ and $a_\text {fs}$ (only) are updated through the scaling factor $C_3$ (Eq. 9) by minimizing $f_{O1}$ again

The overall optimization procedure for the in vivo model is illustrated in the flowchart in Fig. 3.

## Results

### Synthetic model

Table 4 shows the inversely estimated eight parameters from the proposed multi-step optimization procedure with both $f_{vol}$ and $f_\varepsilon$ (Case 1). These are fairly close to the so-called true parameters from [35], though some discrepancy is seen in the values of $a_\text {s}$ and $b_\text {s}$. This is because the objective functions are insensitive to changes in $a_\text {s}$ and $b_\text {s}$.

**Table 4:** Estimated parameters from proposed multi-step optimization procedure

Parameter	True value	Case 1	Case 2	Case 3	Case 4	Parameter range
$a$(kPa)	0.236	0.236	0.236	0.238	0.238	$(0.1, 2)$
$b$	10.81	10.75	10.74	10.61	10.67	$(1, 30)$
$a_\text {f}$(kPa)	20.03	19.96	19.54	19.97	18.97	$(1, 30)$
$b_\text {f}$	14.15	14.38	15.97	15.29	18.45	$(1, 25)$
$a_\text {s}$(kPa)	3.72	3.91	3.83	4.27	4.08	$(0.1, 10)$
$b_\text {s}$	5.16	5.87	6.51	6.99	3.31	$(0.1, 10)$
$a_\text {fs}$(kPa)	0.41	0.41	0.42	0.40	0.43	$(0.1, 2)$
$b_\text {fs}$	11.3	11.52	11.09	12.05	10.28	$(0, 20)$

Case 1: estimation from the proposed optimization procedure

Case 2: estimation using only the strain data in objective function

Case 3: estimation using only the first principal strain and volume data

Case 4: estimation terminated after the first step

Table 4 also shows the results when using $f_\varepsilon$ only for the multi-step optimization procedure (Case 2). Compared to the proposed multi-step optimization (Case 1), the parameters optimized in Case 2 are less accurate, especially for $b_\text {f}$. This highlights the importance of using volume measurements.

Case 3 in Table 4 shows the estimated parameters when we use the proposed multi-step optimization procedure but with only the first principal strains ($k$ = 1st). In this case, the discrepancies in $a_\text {s},\,b_\text {s}$ and $b_\text {fs}$ are greater, though the remaining parameters are still close to the so-called true values. Case 4 arises when all the strains and volume data are used but only the first step of the optimization is performed. The values of $a_\text {f},\,b_\text {f},\,a_\text {s}$ and $b_\text {s}$ are all less accurate in this case. In summary, the proposed multi-step sequential optimization procedure is the best approach of all cases considered.

### In vivo estimation

**Fig. 4:**
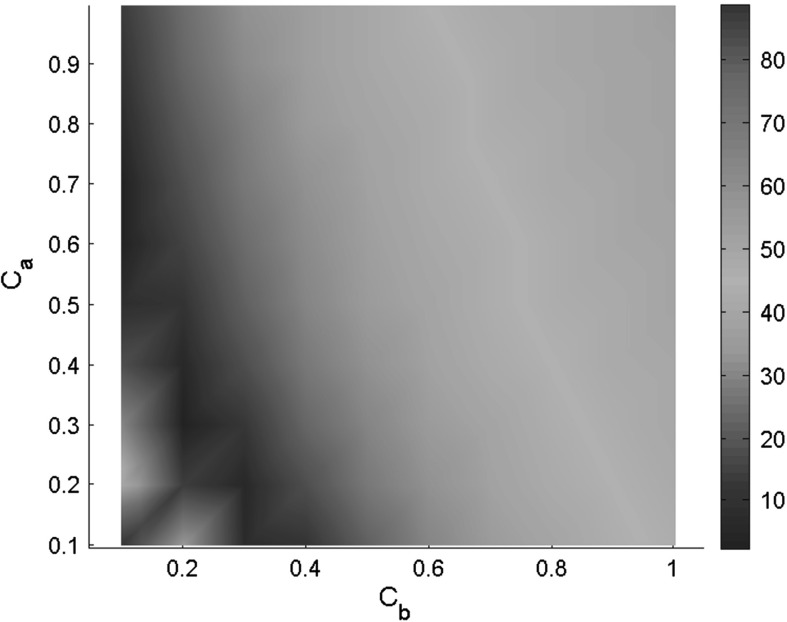
Landscape of objective function of $f_{O1}$ related to $C_a$ and $C_b$

The parameters for the in vivo human LV model are now estimated using the proposed optimization procedure in Fig. 3 with 8 mmHg ED pressure. The mapping of the objective function $f_{O1}$, with $C_a$ and $C_b$ varying in a range of $(0.1 \text{, } 1)$, is shown in Fig. 4. From the centre of the valley, we select the point ($C_a=0.2,\,C_b=0.3$). The final estimated parameters are summarized in Table 5, with the predicted strains compared with the measurements following each step. Clearly, the original parameters used by [35] are over-stiff for the human subjects and result in a much smaller ED LV cavity volume compared to the measured ED volume. This over-stiffness in the parameter set is also noticed in [12].

**Table 5:** Estimated parameters for healthy volunteer

	$a$ (kPa)	$b$	$a_\text {f}$ (kPa)	$b_\text {f}$	$a_\text {s}$ (kPa)	$b_\text {s}$	$a_\text {fs}$ (kPa)	$b_\text {fs}$
Initial	0.2362	10.81	20.0370	14.154	3.7245	5.1645	0.4109	11.3
Step 1	0.0472	3.243	4.0074	4.2462	0.7449	1.5494	0.0822	3.39
Step 2	0.0472	3.243	3.1762	4.7435	0.5426	1.5998	0.0822	3.39
Step 3	0.1348	3.243	3.1762	4.7435	0.5426	1.5998	0.2344	3.39

Volume and strain comparisons	ED volume (mL)		Strain difference		Average strain			
	$\sim$		$\sum _i^n (\varepsilon _i - \varepsilon _i^\mathrm{exp})^2$		$\sum _i^n \varepsilon _i / n$			
Initial	90.57		0.31		$0.06 \pm 0.01$			
Step 1	145.44		0.07		$0.14 \pm 0.04$			
Step 2	150.76		0.06		$0.16 \pm 0.05$			
Step 3	142.99		0.04		$0.18 \pm 0.03$			
Measured	143		$\sim$		$0.17 \pm 0.04$

**Fig. 5:**
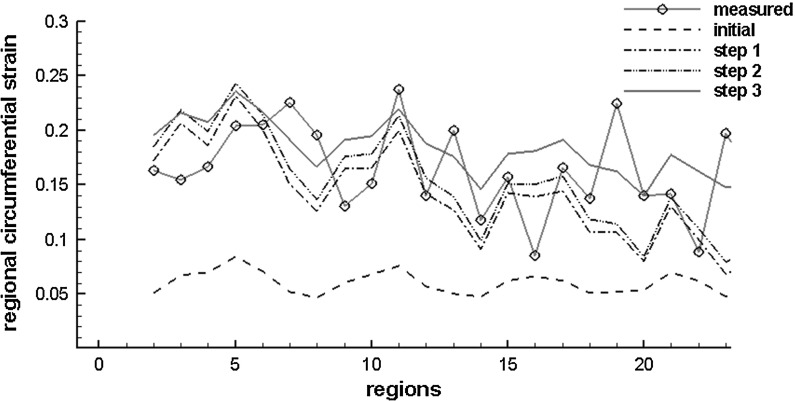
Regional circumferential strain at end of diastole after each optimization step (strain is calculated related to early diastole: the reference state)

**Fig. 6:**
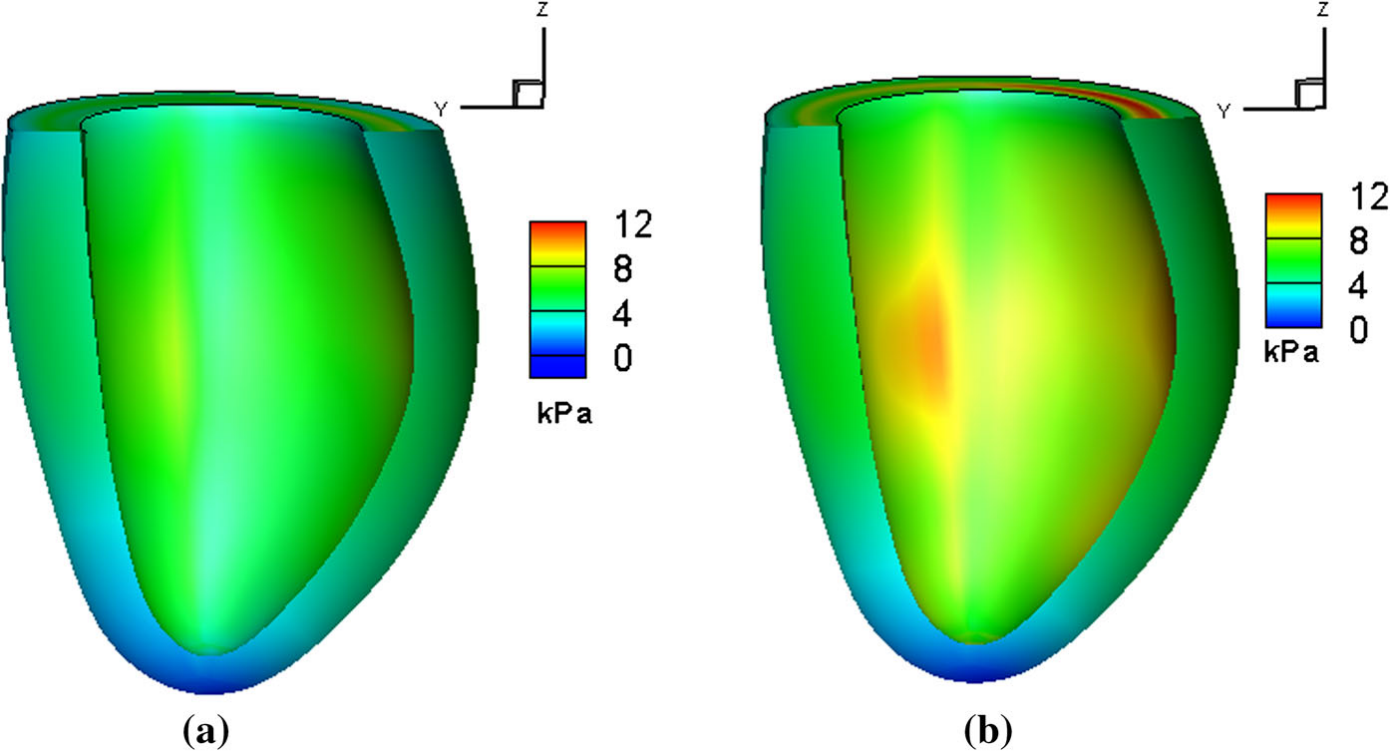
Distributions of myofibre stress at 8 mmHg endocardial pressure for in vivo LV model using **a** initial parameters and **b** optimized parameters

Figure 5 plots the detailed regional circumferential strains at ED after each step. After Step 3, the simulated regional circumferential strain is in good agreement with the measurements. Figure 6 shows the myofibre stress distributions within the LV at ED using both the initial and optimized parameters. Clearly, the initial parameters yield a very different stress pattern compared to the optimized ones.

**Fig. 7:**
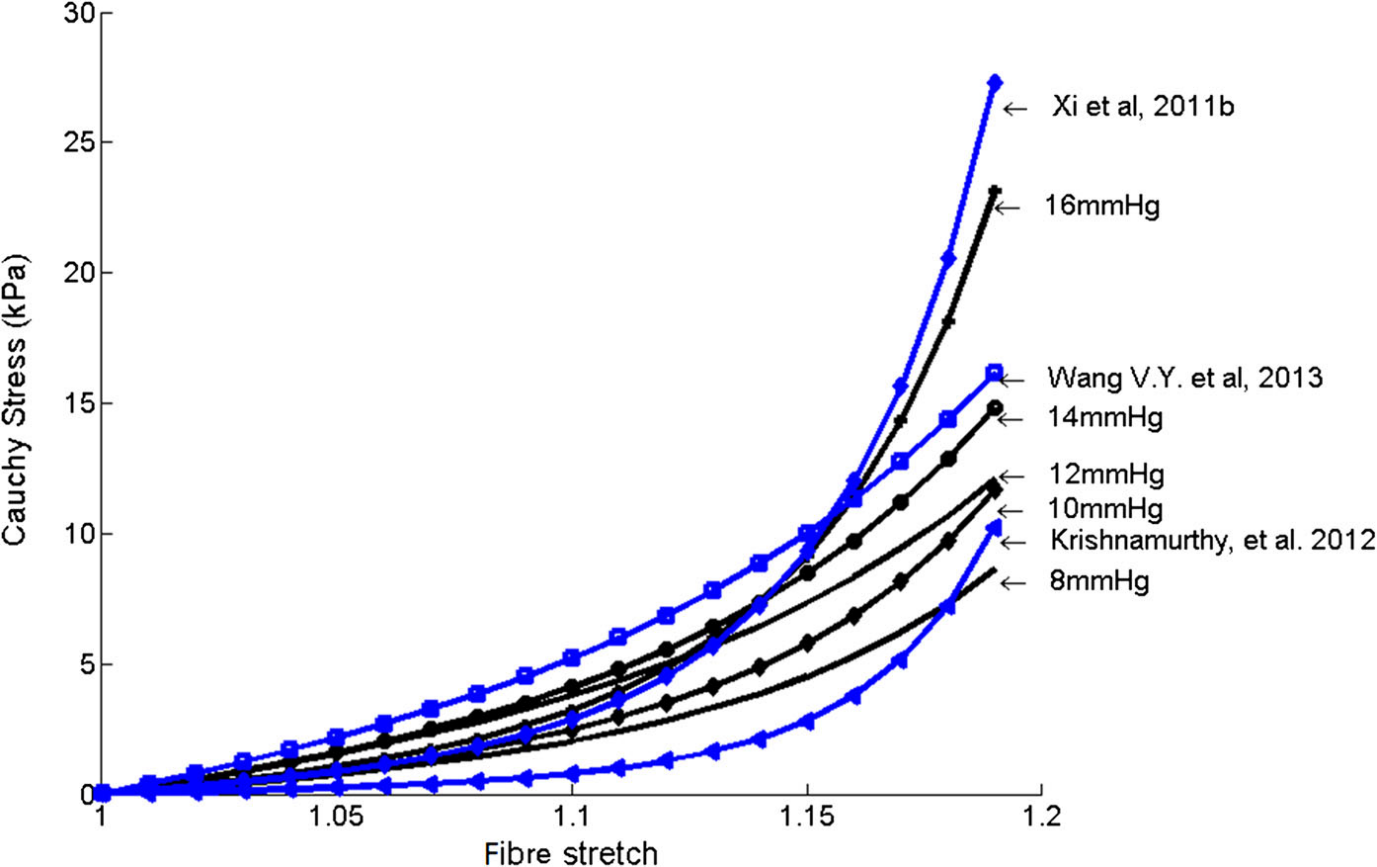
Predicted myofibre stress–strain relationships under uni-axial tension and with parameters estimated from different values of the end-diastolic pressure, compared with predictions from other studies

Since we use a population-based end-diastolic pressure (EDP) in the parameter estimation, we check the change of the parameters when the EDP is varied from 10 mmHg to 16 mmHg; this is summarized in Table 6. Changes in the myofibre stress–strain relationships, when estimated with different EDPs, are plotted in Fig. 7. In general, with increased EDP, the stiffness along the myofibres increases.

**Table 6:** Estimated parameters with different end-diastolic pressures (EDPs)

EDP (mmHg)	$a$ (kPa)	$b$	$a_\text {f}$ (kPa)	$b_\text {f}$	$a_\text {s}$ (kPa)	$b_\text {s}$	$a_\text {fs}$ (kPa)	$b_\text {fs}$
8	0.1348	3.243	3.1762	4.7435	0.5426	1.5998	0.2344	3.39
10	0.0939	4.324	3.7644	5.5372	0.7482	2.0657	0.1634	4.5200
12	0.2658	2.1620	6.4472	2.5886	1.4780	1.0330	0.4625	2.2600
14	0.1211	4.3240	6.8811	3.4370	1.1747	1.7184	0.2107	4.5200
16	0.0509	6.4860	4.2654	8.7982	0.7449	3.0987	0.0885	6.7800

Volume and strain comparisons	ED volume (mL)		Strain difference		Average strain			
	$\sim$		$\sum _i^n (\varepsilon _i - \varepsilon _i^\mathrm{exp})^2$		$\sum _i^n \varepsilon _{i}/n$			
Case with 8 mmHg EDP	142.99		0.04		$0.18 \pm 0.03$			
Case with 10 mmHg EDP	142.99		0.04		$0.18 \pm 0.03$			
Case with 12 mmHg EDP	143		0.04		$0.18 \pm 0.02$			
Case with 14 mmHg EDP	142.99		0.04		$0.18 \pm 0.03$			
Case with 16 mmHg EDP	142.99		0.04		$0.17 \pm 0.04$			
Measured	143		$\sim$		$0.17 \pm 0.04$			

In Fig. 7, we further compare the myofibre stress–strain relationship with those from other studies which inversely estimated human myocardial parameters based on different constitutive laws and EDPs. Xi et al. [42] used a healthy subject and 13.6 mmHg EDP; Wang et al. [29] reported the averaged healthy myocardial stiffness from six subjects and 11 mmHg EDP; Krishnamurthy et al. [12] estimated the functional myocardial stiffness from one LV dysfunctional patient with an EDP of 15 mmHg. Although there are necessary discrepancies due to the subject variety and different constitutive laws used, the overall trend of the mechanical responses is similar.

**Table 7:** Estimated parameters for uncertainty analysis

Case	$a$ (kPa)	$b$	$a_\text {f}$ (kPa)	$b_\text {f}$	$a_\text {s}$ (kPa)	$b_\text {s}$	$a_\text {fs}$ (kPa)	$b_\text {fs}$
$C_a=0.2,C_b =0.3$	0.134	3.243	3.176	4.744	0.543	1.599	0.234	3.39
$C_a=0.17,C_b =0.4$	0.073	4.324	3.072	5.426	0.645	2.007	0.127	4.52
$1.5a_f^\text {ini}$	0.190	2.378	4.018	2.782	0.658	1.132	0.331	2.49
$1.5b_f^\text {ini}$	0.101	4.108	2.236	7.505	0.522	1.962	0.175	4.29
$0.5a_f^\text {ini}$	0.054	4.324	3.182	5.786	1.118	2.065	0.093	4.52
$0.5b_f^\text {ini}$	0.031	5.405	3.718	4.455	0.744	1.797	0.054	5.65
0.5SD Noise	0.147	3.243	2.957	5.104	0.742	1.556	0.255	3.39
1.0SD Noise	0.094	3.243	3.621	4.471	0.772	1.463	0.164	3.39

**Fig. 8:**
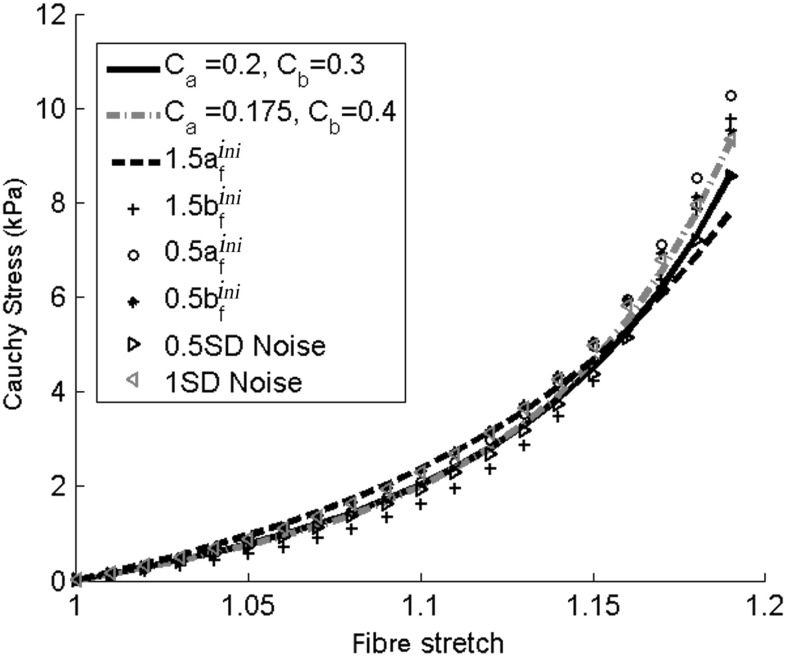
Predicted myofibre stress–strain relationships from different cases listed in Table 7 under uni-axial tension

As for most of the inverse problems, the uniqueness of inversely estimated coefficients cannot be guaranteed. To test the robustness of the procedure, however, we look at seven more cases: (1) using different values of $C_a$ and $C_b$; (2) increasing $a_\text {f}^\text {ini}$ by half; (3) increasing $b_\text {f}^\text {ini}$ by half; (4) decreasing $a_\text {f}^\text {ini}$ by half; (5) decreasing $b_\text {f}^\text {ini}$ by half; (6) adding Gaussian noise to the measured circumferential strains with a half standard deviation of the measured values; and (7) as in (6) but using one standard deviation of the measured values. Table 7 summarizes the estimated parameters from all seven cases, and the corresponding myofibre stress–strain relationships for a cubic myocardial tissue under uni-axial tension are shown in Fig. 8. We notice that although there are some differences in the estimated parameters for the different cases, there seems to be reasonable agreement in the predicted myofibre stress–strain relationships (Fig. 8). This is confirmed by applying the optimization procedure to two more healthy volunteers (see the appendix for details). The myofibre stress distributions obtained using different sets of parameters in Table 7 are all found to be similar to the stress distribution in Fig. 6b (and hence not shown). Since the myofibre stress–strain relationship largely determines the LV responses, and given the extreme difficulty in the in vivo parameter estimation, the proposed optimization procedure is deemed to be sufficiently robust.

## Discussion

Estimating the material parameters of myocardial constitutive laws from limited in vivo data remains a major challenge due to the non-linearity of the model responses and strong intercorrelations between the material parameters [2]. Furthermore, different constitutive laws have different parameter sets and behaviours, and specific treatment in the inverse problem is often required for a given constitutive law. Although the structure-based Holzapfel–Ogden law is gaining popularity in heart modelling, to the best of our knowledge, no studies have been performed to estimate the parameters of the Holzapfel–Ogden law from in vivo measurements.

In this study, inspired by the ideas from previous studies [20, 27, 30], we first developed an optimization procedure for estimating the parameters of the Holzapfel–Ogden law using a synthetic model to produce the so-called true values. The synthetic model allows us to investigate the estimation errors and verify the correctness of the solution. By performing the sensitivity analysis, we show that even with a large amount of strain and pressure–volume data, it is difficult to accurately estimate the sheet parameters $a_\mathrm{s}$ and $b_\mathrm{s}$, which are usually very small [20]. We also find that by using a multi-step and sequential optimization procedure we can achieve much higher accuracy compared to a single-step optimization (Table 4).

When applying the approach to in vivo LV models, we have encountered difficulties since there are insufficient data from clinical measurements. Various constraints must be introduced to reduce the complexity of the problem. One constraint is to assume that the eight parameters can be grouped into two (one with exponential terms, and one without) and scaled to the corresponding groups of the original parameters [35]. In this way, we only need to estimate two scaling factors, $C_a$ and $C_b$, in the first step. In addition, the set which minimizes $|C_a-C_b|$ is chosen when multiple sets of $C_a$ and $C_b$ are available from the sweeping procedure. This also avoids over-stiffness responses. A similar approach is used by [12], though for a different constitutive law.

Furthermore, constraints (Eq. 8) must be introduced when estimating $a_\text {f}$ and $b_\text {f}$ using normalized volume and strain data. Because the measured circumferential strain from cine images is in the circumferential–longitudinal axis plane, which aligns with the myofibre direction, this procedure can effectively update the myofibre stiffness parameters, $a_\text {f}$ and $b_\text {f}$. However, we are unable to accurately estimate $a_\text {s}$ and $b_\text {s}$, which are related to myofibre sheet strains. Measurements of sheet strains are extremely difficult in vivo. In addition, the synthetic model study shows that even with all the principal strains, $a_\text {s}$ and $b_\text {s}$ have very low sensitivities and cannot be estimated properly. Hence, additional constraints are applied to $a_\text {s}$ and $b_\text {s}$ in the second optimizing step.

The sensitivity analysis shows that the parameters in the Holzapfel–Ogden law are highly intercorrelated; for example, an increase in $a$ can be compensated by a decrease in $b$ or other correlated parameters. This leads to uncertainties in the parameter estimation. This is a common issue for all anisotropic constitutive laws. Xi et al. [2] found that, with their synthetic LV models, a unique solution for estimating the seven parameters of Costa’s law [33] cannot be achieved using a reduced-order unscented Kalman filter. Therefore, they used the transversely isotropic Guccione’s law with a lower level of complexity.

Instead of changing the constitutive law, we reduce the complexity of the problem by estimating a total of five parameters only, $C_a,\,C_b,\,a_\text {f},\,b_\text {f},\,C_3$. Even with the reduced set of parameters, it is not possible to establish the global minimization or the uniqueness of the solution given the ill-posed nature of the inverse problem. However, we have tested various cases to show that more or less the same mechanical responses in the physiological range could be achieved even though the parameters are somewhat different. One possible explanation for the same stress–strain relationship from different parameter values of the same constitutive law is that the law is based on coupled strain attributes [43].

In this study, only cine CMR imaging is used to provide data for the in vivo LV model. Although dedicated strain CMR imaging is able to provide 3D LV deformation, this requires complex image processing and additional scanning time, which may not be possible for some patients. Cine CMR images are widely available from routine scans. Hence the optimization procedure proposed based on cine CMR imaging can be readily used for clinical applications. The downside of the approach is that accurately estimating LV motion/strain from cine CMR images is more difficult because cine images are usually 2D images; therefore, the out-plane motion cannot be easily estimated. In addition, due to the lack of patterns or features for motion tracking, large uncertainties exist when estimating the pixel-wise strain. Our previous study [41] showed that regional circumferential strains estimated from cine images using a deformable image registration method compared well with those from dedicated strain CMR imaging for both healthy volunteers and patients with myocardial infarction, but greater discrepancies existed in the estimated regional radial strains. Thus, in the in vivo LV model, only regional circumferential strains are used for the objective function.

The dilemma is that, while fewer data make the inverse problem more ill-posed, demanding more data means more clinical measurements with longer acquisition times, which is not always possible. In a patient CMR study, only necessary measurements are performed routinely. Furthermore, CMR image at late-diastole is difficult to achieve, while the ED frame is always recorded with high quality; therefore, it is desirable to use information from the ED frame for material parameter estimation. However, issues associated with inversely estimating material properties from an ED frame have been reported in several studies [26]. Xi et al. [42] found that with an ED measurement, the parameters cannot be uniquely constrained, although such a measurement can provide a potentially robust indicator of myocardial stiffness, which is similar to what we found. Augenstein et al. [25] showed that with five frames of CMR data, the parameters from Guccione’s law could be inversely estimated within a 5% margin of error.

In our study, because of the lack of an in vivo EDP recording and strain CMR, we must rely on the limited ED frames for the parameter estimation. Therefore, rather than aiming to obtain the unique parameters, which is not possible, we try to extract the myocardial stiffness. It goes without saying that with more data available from routine CMR measurements, the parameters could be estimated with higher accuracy. This has been demonstrated by the synthetic model: the inversely estimated parameters are fairly close to the so-called true parameters.

In the in vivo LV model, an original set of parameters is required. We used a set obtained by fitting the simple shear tests on healthy swine myocardial samples [15, 35]. These original parameters might affect the accuracy of inverse estimation. Parameters based on human myocardial samples may be a better candidate, but these are not yet available.

Validating the inversely estimated parameters in the in vivo LV model is difficult since it is impossible to perform mechanical tests on in vivo hearts. Previous studies tried to compare results with those of other studies [2, 28]. Our estimated parameters are shown to be in the same range as in [12, 29, 42].

An important issue in LV modelling is the choice of a suitable constitutive law for the myocardium. In this paper, we choose the well-established Holzapfel–Ogden model. We are aware that the Holzapfel–Ogden law makes no use of the deformation invariants $I_5 (=\mathbf{f_0 C}^2\mathbf{f_0}))$ and $I_7 (=\mathbf{s_0 C}^2\mathbf{s_0})$, which has recently been shown to be incompatible with linear elasticity [44, 45]. However, it is well known that if you reduce the number of invariants, then you cannot fully capture linearly elastic responses, but the consequences of this should be viewed with extreme caution since experiments on soft tissue in the small-strain regime, by the very nature of the material, are not very accurate or reliable. Indeed, many of the current invariant-based laws do a very good job of fitting the data of a wide range of soft tissues to a wide range of deformations – and the fact that they do not fully capture linear elasticity is, under the present state of experimental knowledge of the mechanics of soft tissues, largely irrelevant. The Holzapfel–Ogden law, in particular, is the model that can fit all the data of the simple shear experiments well [15], and the computed LV dynamics based on the Holzapfel–Ogden law seem to predict the measured strains and volumes well within the physiological range. In addition, including more invariants will greatly increase the complexity of the inverse problem and make it extremely hard, if not impossible, to estimate all the parameters from in vivo measurements.

Other limitations of the work in common with many published LV models are as follows: (1) the in vivo LV geometry is reconstructed at ED, which is not stress-free, and residual stress is not considered [39]; (2) the heterogeneous distribution of material properties is not considered in the LV models; and (3) the proposed method can be potentially extended to diseased heart tissue with some changes in the optimization procedure to account for a remodelled micro-structure, such as myocardial infarction [46].

## Conclusion

In this study, we have investigated, for the first time, the feasibility of inversely estimating parameters in the orthotropic Holzapfel–Ogden constitutive law for passive myocardium by proposing a multi-step optimization procedure using both strain and pressure–volume data. When applied to a synthetic LV model, the estimated parameters are very close to the known parameters, although some uncertainties exist in estimating parameters along the sheet direction due to the low sensitivity. For parameter estimation of in vivo models, a more comprehensive sensitivity study is performed due to the limited measurement data. The material parameters are scaled from the original parameters based on ex vivo experimental tests. A study of the sensitivity is also used to reduce the complexity of the problem. By matching the regional circumferential strains and pressure–volume at the ED frame, we have demonstrated that the parameters of the Holzapfel–Ogden law, and in particular the myofibre stress–strain relationship, can be estimated successfully when suitable constraints are introduced for the in vivo model.

## Figures and Tables

**Fig. 9:**
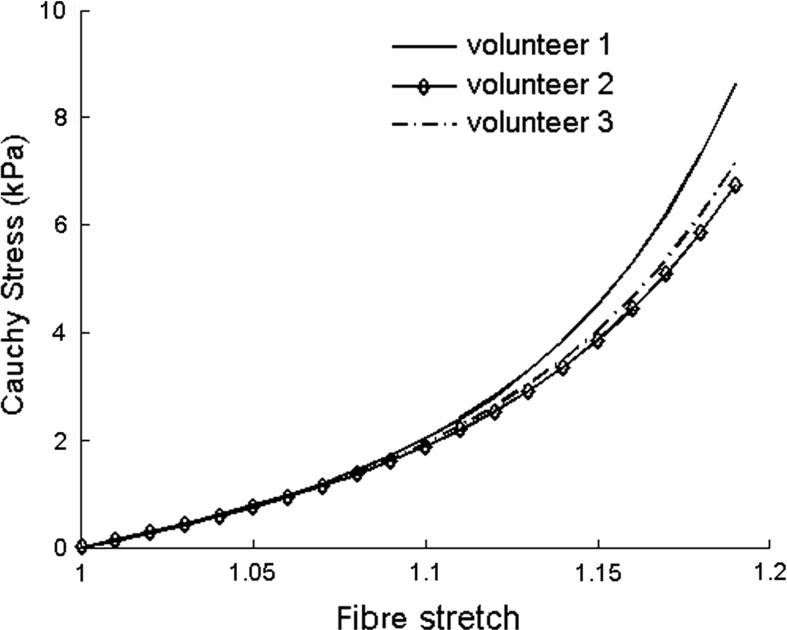
Myofibre stress–strain relationships under uni-axial tension for three healthy volunteers

## Appendix

The proposed optimization procedure is further applied to two more healthy volunteers [volunteer 2 (male, age 22) and volunteer 3 (male, age 31)]. The LV geometry is reconstructed from CMR studies similarly to the volunteer study in the main text (volunteer 1, male, age 28), and 8 mmHg EDP is assumed. Table 8 contains the inversely estimated parameters of the Holzapfel–Ogden law. Parameters are similar for all three volunteers, though minor differences exist. Table 9 compares the ED regional circumferential strain in the three volunteers with strains from cine images, which shows that with optimized parameters, the simulated strains agree well with the measurements. Figure 9 in Appendix shows the corresponding myofibre stress–strain relationships for all three volunteers under uni-axial tension. Again, the mechanical responses for these healthy volunteers are very similar, particularly at the small strain (Fig. 9).

**Table 8:** Estimated parameters from three healthy volunteers with 8 mmHg EDP

	$a$ (kPa)	$b$	$a_\text {f}$ (kPa)	$b_\text {f}$	$a_\text {s}$ (kPa)	$b_\text {s}$	$a_\text {fs}$ (kPa)	$b_\text {fs}$
Volunteer 1	0.1348	3.243	3.1762	4.7435	0.5426	1.5998	0.2344	3.39
Volunteer 2	0.2096	3.243	3.0634	3.4595	0.7334	1.5473	0.3646	3.39
Volunteer 3	0.1034	3.243	3.2205	3.5845	0.7418	1.5470	0.1799	3.39

**Table 9:** ED regional circumferential strain comparison

	Measured	Inversely estimated
Volunteer 1	$0.17 \pm 0.04$	$0.18 \pm 0.03$
Volunteer 2	$0.19 \pm 0.06$	$0.19 \pm 0.04$
Volunteer 3	$0.19 \pm 0.05$	$0.20 \pm 0.04$
